# A pH probe inhibits senescence in mesenchymal stem cells

**DOI:** 10.1186/s13287-018-1081-0

**Published:** 2018-12-07

**Authors:** Lihong Wang, Xianjing Han, Guojing Qu, Le Su, Baoxiang Zhao, Junying Miao

**Affiliations:** 1 Shandong Provincial Key Laboratory of Animal Cells and Developmental Biology, School of Life Science, Shandong University, Jinan, 250100 People’s Republic of China; 2 Institute of Organic Chemistry, School of Chemistry and Chemical Engineering, Shandong University, Jinan, 250100 People’s Republic of China; 3 The Key Laboratory of Cardiovascular Remodeling and Function Research, Chinese Ministry of Education and Chinese Ministry of Health, Shandong University Qilu Hospital, Jinan, 250012 People’s Republic of China

**Keywords:** Autophagy, Bafilomycin-A1 antagonist, Senescence, Bone marrow-derived mesenchymal stem cells

## Abstract

**Background:**

Bone marrow-derived mesenchymal stem cells (BMSCs) are gradually getting attention because of its multi-directional differentiation potential, hematopoietic support, and promotion of stem cell implantation. However, cultured BMSCs in vitro possess a very limited proliferation potential, and the presence of stem cell aging has substantially restricted the effect together with the efficiency in clinical treatment. Recently, increasing attention has been paid to the connection between cellular aging and lysosomal acidification as new reports indicated that vacuolar H^+^-ATPase (v-ATPase) activity was altered and lysosomal pH was dysregulated in the process of cellular aging. Therefore, promoting lysosomal acidification might contribute to inhibition of cell senescence. Our previous studies showed that a novel small molecule, 3-butyl-1-chloro imidazo [1, 5-a] pyridine-7-carboxylic acid (SGJ), could selectively and sensitively respond to acidic pH with fast response (within 3 min), but whether SGJ can promote lysosomal acidification and inhibit senescence in BMSCs is unknown.

**Methods:**

Rat BMSCs were cultured based on our system that had been already documented. BMSCs were treated with SGJ and/or Bafilomycin-A1 (Baf-A1). The co-localization between SGJ and lysosomes was assessed by a confocal microscope. Acridine orange (AO) staining and the Lysosensor™ Green DND-189 reagents were used for indicating changes in lysosomal concentration of H^+^. Changes of senescence were detected by immunoblotting of p21 and senescence-associated beta-galactosidase (SA-β-gal) staining as well as immunofluorescence assay of senescence-associated heterochromatin foci (SAHF). Changes of autophagy were detected by immunoblotting of MAP1LC3 (LC3B) and SQSTM1 (p62). Cell proliferation was determined by flow cytometry. Cell viability was calculated by sulforhodamine B assay (SRB). The V0 proton channel of v-ATPase was knocked down by transfecting with its small interfering RNA (si-ATP6V0C).

**Results:**

Our work showed that SGJ can promote lysosomal acidification and inhibit senescence in BMSCs. Firstly, SGJ and lysosomes were well co-located in senescent BMSCs with the co-localization coefficient of 0.94. Secondly, SGJ increased the concentration of H^+^ and the protein expression of lysosome-associated membrane protein 1 (LAMP1) and lysosome-associated membrane protein 2 (LAMP2). Thirdly, SGJ suppressed the expression of p21 in the senescent BMSCs and reduced SA-β-gal positive cells. Fourthly, SGJ promoted senescent BMSCs’ proliferation and protein level of LC3B but reduced the p62/SQSTM1 protein level. Furthermore, experimental group pretreated with 20 μM SGJ showed a stronger red fluorescent intensity, thinner cell morphology, less SA-β-gal positive cell, and less p21 protein level as well as higher cell viability in the presence of Baf-A1. Notably, ATP6V0C knockdown decreased the activity of v-ATPase and SGJ increased the concentration of H^+^.

**Conclusion:**

Our work showed that SGJ could inhibit senescence in BMSCs and protect lysosomes by promoting expression of LAMP1 and LAMP2. Meanwhile, SGJ could promote autophagy. Furthermore, our study also suggested that SGJ was a new Baf-A1 antagonist because SGJ could target and occupy the V0 proton channel of v-ATPase.

**Electronic supplementary material:**

The online version of this article (10.1186/s13287-018-1081-0) contains supplementary material, which is available to authorized users.

## Background

An important member of stem cell family, bone marrow-derived mesenchymal stem cell (BMSC) that can be induced to differentiate into adipocytes, osteocytes, muscle cells, nerve cells and endothelial cells, originated from the early mesoderm, are gradually getting attention because of its multi-directional differentiation potential, hematopoietic support and promotion of stem cell implantation [1–3]. The application of BMSCs in clinical infusion is now providing a great hope for cell therapies and tissue engineering in a wide variety of diseases [4–7]. We think that BMSCs can be used as an ideal seed cell for aging and lesions in the future [8, 9]. However, cultured BMSCs in vitro possess a very limited proliferation potential, and the presence of stem cell aging has substantially restricted the effect together with the efficiency in clinical treatment, especially impeding the development of an autologous cell-based therapy for senior patients [10].

Cellular senescence involves an extensive range of blocking cellular remodeling and cycling, including chromatin structure [11–13]. It has been suggested that the increased intracellular balance failure during cell aging was partly due to the impaired function of lysosome that was the key organelle for autophagy, and the presence of lysosome and autophagy was important to cellular constituents recycling and cell remodeling involved in cellular senescence [1, 14–18]. In addition, metabolites and ions released by lysosomes play a vital role in signal transduction and nutritional sensation, which are affected by intraluminal pH of lysosomes [19, 20]. Lysosomes maintain an acidic lumen by means of the proton pump and the vacuolar H^+^-ATPase (v-ATPase) [21]. H^+^-ATPases are large, multi-subunit complexes composed of a peripheral domain (V1) that hydrolyzes ATP and a membrane integral domain (V0) that translocates protons into lysosomes and therefore lowers the intraluminal pH to the acidic range [22]. With the acidic pH optimum conditions, dozens of hydrolytic enzymes in the lysosome are activated [23]. Therefore, the disorders of lysosomal acidification can have obvious effects on lysosomal digestion. The disorders strongly inhibit the activity of acidic hydrolases, but they also potentially promote the activities of other neutral hydrolases [24]. This transformation will promote both substrate indigestion and atypical cleavages, which may also produce toxic products and/or partially digested intermediates [1]. Increasing attention has been paid to the connection between cellular aging and lysosomal acidification as new reports are indicating that v-ATPase activity is altered and lysosomal pH is dysregulated in the process of cellular aging [1]. The disorders of lysosomal acidification increase the risk of cellular aging and death [1]. However, vacuolar acidification appears to be critical in mediating the prolonged lifespan effects of caloric restriction and methionine restriction [25–27]. In addition, overexpressing of the vacuolar ATPase (v-ATPase) components promotes an increase in lifespan in the yeast models [25, 27]. Increased vacuolar ATPase (v-ATPase) early in life contributes to age-related mitochondrial dysfunction. It is reasonable to expect that upregulated function of v-ATPase that promotes lysosomal acidification contributes to inhibition of cell senescence [25–27]. Thus, successful applications of small chemical molecules in inhibiting senescence in BMSCs by maintaining lysosomal functions provide potential intervention strategies to prevent body from aging and regenerate tissues [28]. Our previous work showed that a novel small molecule, 3-butyl-1-chloro imidazo [1, 5-a] pyridine-7-carboxylic acid (SGJ), had been demonstrated as a fluorescent probe to selectively and sensitively respond to acidic pH with fast response (within 3 min) [29]. Here, we wish to report our studies on SGJ, in which clarify its functions in lysosomes, relationship with Baf-A1 and role in suppressing cell senescence.

## Methods

### Cell extraction and culture

Rat bone marrow collection and mesenchymal stem cell isolation were performed according to Pittenger et al. In brief, bone marrow in the femurs and tibias of male Wistar rats (90–100 g) was washed by using Dulbecco’s modified Eagle’s medium-low glucose (DMEM-LG) (Gibco) supplemented with 10% fetal bovine serum (HyClone). BMSCs were isolated by removal of non-adherent cells after 72 h, and the medium was changed every 2 days until the adherent cells reached 50% confluence, and they were then harvested with 0.05% trypsin (Sangon Biotech) in phosphate-buffered saline (PBS). Cells were grown to 70–80% confluence and then seeded on plates or appropriate dishes at the density of 5000 cm^− 2^. The experiments were conducted on cells at varied population doubling level (PDL) to demarcate young (PDL 3–6) and senescent (PDL 16–25) BMSCs.

### Cell treatment

BMSCs were cultured in Dulbecco’s modified Eagle’s medium-low glucose (DMEM-LG) (Gibco) supplemented with 10% fetal bovine serum (HyClone) at 37 °C in a humidified incubator with 5% CO_2_. In the process of the whole experiments, the cell’s density was around 90% before doing all experiments, and the decentralized cells were seeded onto 6-well plates, 12-well plates, 24-well plates, 96-well plates, or other appropriate dishes at the density of 20,000 cells/mL with pre-incubation for 12 h before adding the compound SGJ. SGJ was synthesized as reported with a purity greater than 98% and the structure was confirmed by spectral data. SGJ was dissolved in dimethylsulfoxide (DMSO) to make a 0.1 M stock solution. BMSCs were incubated with culture medium containing 1, 5, 10, and 20 μM SGJ, or a corresponding volume of DMSO solvent as control (below 0.1%, *v*/v) in all subsequent experiments.

### Cell morphological observation

BMSCs were seeded onto 24-well plates and treated with 0.1% DMSO as a negative control or 1, 5, 10, and 20 μM SGJ for 24 or 48 h, respectively, and the microscopic photographs (×200) were taken under the inverted phase-contrast microscope (Nikon, Japan).

### Cell viability assay (SRB)

Referring to the relevant reports, BMSCs were seeded onto 96-well plates. After 12 h, cells were respectively treated with 0.1% DMSO as a relevant control or compound SGJ at 1, 5, 10, and 20 μM for specified time durations. Then the cell viability was evaluated by sulforhodamine B assay (SRB), according to the method of Skehan. In short, discarded the original medium and added 100 μL cold 10% trichloroacetic acid (TCA) into the 96-well plates to fix cells and incubated for 1 h at 4 °C. Poured off the supernatant and washed the cell five times with ultrapure water. After drying the 96-well plates at room temperature, we added 50 μL of 0.4% (*w*/*vW*/*V*) SRB solution in 1% acetic acid into each hole and shook it for 10 min on a microtiter plate shaker. And then we washed the 96-well plates five times with 1% acetic acid and subsequently added 100 μL of 10 mM Unbuffered Tris Base (pH = 10.5) to dissolve the original dye after again drying the 96-well plates at room temperature. We shook the plate on a microtiter plate shaker for 10 min and measured the light absorption at the wavelength of 540 nm by a SpectraMAX190 microplate spectrophotometer (GMI Co, USA).

### Flow cytometry

Cells were digested into single cells by 0.25% trypsin (Sangon Biotech) and collected into 15-mL centrifuge tubes. Centrifugation at 1000 rpm for 5 min was performed, then we discarded the supernatant. We washed cells twice with 1× PBS buffer and suspended them in 1× PBS buffer by centrifugation at 1000 rpm for 5 min each time. We discarded the supernatant. We suspended cells by 1 mL cold 70% ethyl alcohol and fixed them at 4 °C overnight. We washed cells three times with 1× PBS buffer by centrifugation at 1000 rpm for 5 min each time. We discarded the supernatant and suspended 1 × 10^6^ or 1 × 10^7^ cells with 500 μL 1× PBS buffer including 100 μg/mL propidium iodide (PI) (Biolegend), 50 μg/mL RNAase (TIANGEN), and 0.2% X-Triton 100 (Sangon Biotech), and placed it at 4 °C for 30 min. It was analyzed by flow cytometry.

### Senescence-associated β-galactosidase assay

BMSCs seeded in 24-well plates were exposed to 2% formaldehyde/0.5% glutaraldehyde for fixation. After 5 min, cells were rinsed with PBS and warmed β-gal buffer solution (5 mM potassium ferrocyanide, 5 mM potassium ferricyanide, 150 mM NaCl, and 2 mM MgCl_2_, pH = 6.0) for 1 min. Then, cells were incubated with staining solution (buffer solution supplemented with 1 mg mL^− 1^ 5-bromo-4-chloro-3-indolyl β-_D_-galactopyranoside, Takara) for over 18 h at 37 °C. Senescent cells were stained to be blue under a phase-contrast microscope. The percentage of positively stained cells was estimated by counting at least 1500 cells in each sample.

### Lysosomal activity detection

Lysosomal activity was monitored by a metachromatic fluorophore acridine orange (AO) (0.1 mg mL^− 1^). After treatment, BMSCs were gently rinsed twice with PBS and then subjected to acridine orange staining for 1 min. Cells were washed with PBS and the fluorescence was observed under an inverted fluorescence microscope (Nikon).

### Immunofluorescence microscopy

For the next immunofluorescence assay, we seeded BMSCs onto confocal dishes (20 mm) (SPL, Korea) and used SGJ to treat cells. After treatment, we aspirated the culture medium and rinsed the cells gently with 1× PBS, then the cells were fixed in 4% paraformaldehyde for 15 min. After washing three times with 1× PBS, permeated cells with 0.1–0.2% TritonX-100 for 5 min, then washed and blocked with donkey serum (1:30 dilution in 0.1 M PBS) for 20 min at room temperature. We discarded the enclosed liquid, incubated cells with primary HP1-γ antibody (Proteintech), H3K9Me2/3 antibody (Cell signaling, USA) (1:100 dilution) overnight at 4 °C, then incubated cells with Alexa 488 nm labeled species-specific secondary antibodies (1:200 dilution in 0.1 M PBS) for 60 min at 37 °C. Finally, we rinsed and stained cells with DAPI and monitored them by a confocal laser scanning microscope (Zeiss, Germany).

### GFP-LC3B puncta observation

U87 cells were seeded onto confocal dishes (20 mm) (SPL, Korea) and used SGJ and chloroquine (CQ) to treat cells. After treatment, we discarded the culture medium and rinsed the cells gently with 1× PBS, then the cells were fixed in 4% paraformaldehyde for 15 min. After washing three times with 1× PBS, we observed the cells with a confocal laser scanning microscope (Zeiss, Germany).

### Western blot analysis

As described previously, cells were treated with 0.1% DMSO or SGJ for specific time and washed twice with ice-cold PBS, then lysed in protein lysis buffer (Shanghai Beyotime Co, China) that contained 0.5% SDS in 25 mM Tris–HCl, 4 mM EDTA, 100 mM NaCl, 1 mM PMSF, 10 μg mL^− 1^ leupeptin, and 10 μg mL^− 1^ soybean trypsin inhibitor, pH = 7.5. The protein concentrations were determined by the Bradford method. Before being loaded into a 12% or 15% SDS polyacrylamide gel, equal amounts of proteins were added with loading buffer and boiled. Following electrophoresis, the resolved protein was electrophoretically transferred to a polyvinylidene fluoride (PVDF) membrane (Millipore, MA, USA). The membrane was blocked with 5% non-fat milk in TBST (Tris buffer saline containing 0.5% Tween 20) for 1 h at room temperature. Subsequently, the membrane was probed with p21 antibody (1:2000) (Proteintech, USA), LC-3B antibody (1:2000) (Cell signaling, USA), p62 antibody (1:2000) (MBL, Japan), LAMP1 antibody (1:500) (BOSTER, USA), LAMP2 antibody (1:1000) (Santa, USA), or anti-β-actin mouse monoclonal antibody (1: 2000) (SIGMA, USA) overnight at 4 °C, then it was washed with TBST for 5 min twice. The membrane was subsequently incubated with HRP-conjugated goat anti-rabbit IgG (1:5000) (Beijing Dingguo Co, China) or polyclonal goat anti-mouse immunoglobulins/HRP (1:5000) (Beijing Dingguo Co, China) for 1 h at room temperature and then washed three times with TBST. Then the membrane was incubated with HRP substrate for 5 min, and the fluorescence signal was detected with X-ray films. Intensity of the protein bands was quantified by Quantity-One software (Bio-Rad), analyzed by ImageJ software, and normalized to loading controls.

### Lysosomal co-localization experiment

We seeded BMSCs onto confocal dishes (SPL, Korea). After 12 h, cells were treated with 0.1% DMSO (as control) or compound SGJ (20 μM) for 1 h, and then they were treated with 0.5 μM LysoTracker® Red DND-99 for 30 min. We washed cells with 1× PBS for three times and monitored them by a confocal laser scanning microscope (Carl Zeiss, Germany). The excitation wavelength of SGJ is 380 nm, while the excitation wavelength of LysoTracker® Red DND-99 is 577 nm.

### Lysosomal pH sensing experiment

We seeded BMSCs onto confocal dishes (SPL, Korea). After 12 h, the cells were treated with 0.1% DMSO (as control), compound SGJ (20 μM), and/or Bafilomycin-A1 (Baf-A1) (20 nM) for a specific time, and then cells were treated with 0.5 μM Lysosensor™ Green DND-189 (pH = 5.2) for 30 min. We washed cells with 1× PBS three times and monitored them by a confocal laser scanning microscope (Carl Zeiss, Germany). The excitation wavelength of Lysosensor™ Green DND-189 is 443 nm.

### Si-RNA interference experiment

The specific siRNA against ATP6V0C was synthesized by Invitrogen. Scramble siRNA was used as a control (Santa Cruz, sc-37007). The corresponding negative control (NC) was designed and purchased from Invitrogen. Cells at 70% confluence were transfected with siRNA by Lipofectamine 2000 (Invitrogen, 11668–019) transfection reagent according to the manufacturer’s instructions.

### Statistical analyses

Data were presented as means ± SE from no less than three independent experiments and analyzed by SPSS (Statistical Package for the Social Sciences) software and Student’s *t* test. Pictures were processed with Adobe Photoshop software. The mean values were derived from at least three independent experiments. Differences at *p* < 0.05 were considered statistically significant.

## Results

### SGJ co-located with lysosomes

**Fig. 1:**
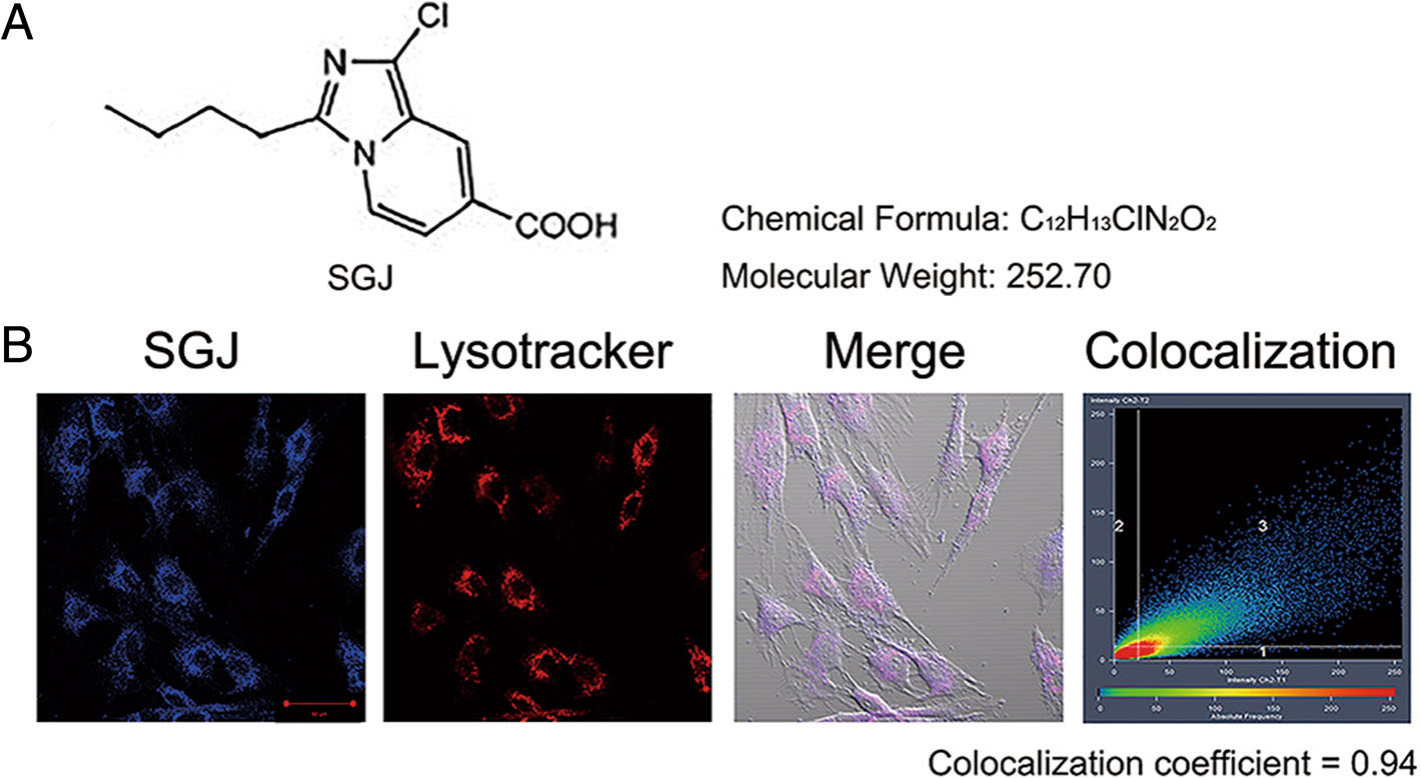
SGJ co-located with lysosomes. **a** The chemical structure of SGJ, 3-butyl-1-chloro imidazo [1, 5-a] pyridine-7-carboxylic acid. **b** Lysosomal co-localization experiment. BMSCs were treated with 0.1% DMSO (as control) or 20 μM SGJ for 1 h, and then treated cells with 0.5 μM LysoTracker® Red DND-99 for 30 min. Monitored the red and blue fluorescence by a confocal laser scanning microscope, and calculated the co-localization coefficient is 0.94

The chemical structure of the small molecule SGJ is shown in Fig. 1a. To explore the relation between SGJ and lysosome, we treated BMSCs with SGJ and LysoTracker® Red DND-99. We found that SGJ and lysosome are well co-located in senescent BMSCs with the co-localization coefficient of 0.94 (the complete co-localization coefficient is 1) (Fig. 1b).

### SGJ increased the concentration of H^+^ and protected the function of lysosomes in senescent BMSCs

**Fig. 2:**
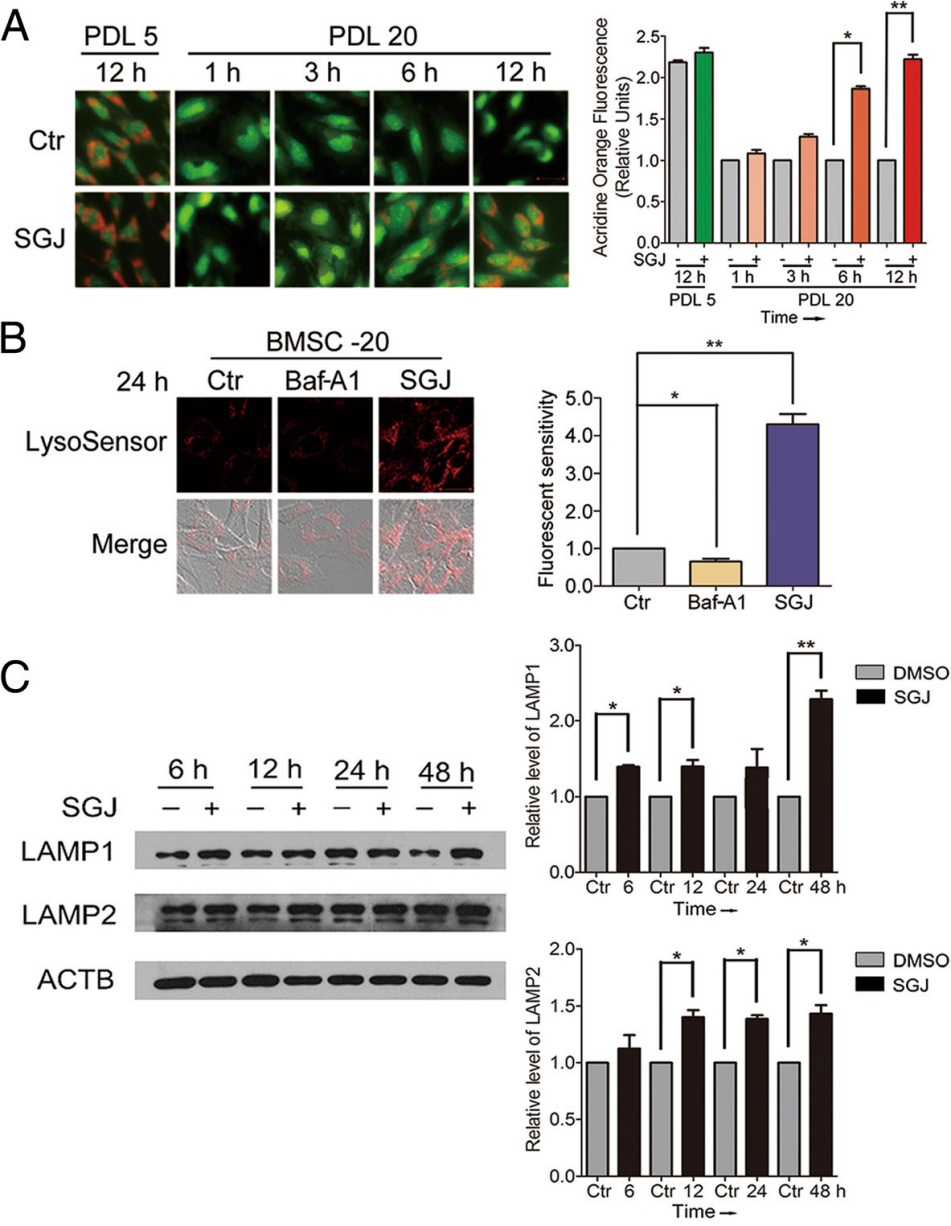
SGJ increased the concentration of H^+^ in lysosomes, and up-regulated LAMP1 and LAMP2 protein level. **a** Acridine orange staining for young (PDL 5) and senescent (PDL 20) BMSCs. Acidic vacuoles declined with age as shown in the results. Twenty-micromolar SGJ treatments for 1, 3, 6, and 12 h significantly restored the amount of acidic vacuoles (magnification × 200). **b** SGJ promoted lysosomal acidification. Lysosensor™ Green DND-189 was used to sense the changes of the concentration of H^+^ in lysosomes, and quantification. BMSCs were treated with 20 nM Baf-A1 or 20 μM SGJ for 24 h. The changes of the red fluorescence reflect changes in lysosomal pH. **c** Western blot analysis of LAMP1 and LAMP2 protein levels with β-actin as a loading control, and quantification. BMSCs were treated with 20 μM SGJ for 6, 12, 24 and 48 h. (*, *p* < 0.05; **, *p* < 0.01, results were expressed as means ± SEM, *n* = 3)

Wang et al. proved that lysosomal activity declined and acidic vacuoles decreased with age [28]. Acridine orange (AO) is normally used as an indicator for changes in lysosomal pH, lysosomal integrity and permeability [30, 31]. As shown in Fig. 2a, to clarify the function of SGJ in lysosome, we performed AO staining. The results showed that the senescent cells at PDL 20 displayed a dim red fluorescence compared to the young cells at PDL 5. SGJ treatment greatly increased red puncta in the senescent cells, indicating a higher level of acidic vacuoles. To investigate whether SGJ functioned by increasing the concentration of H^+^ in the senescent lysosome or not, we next utilized Baf-A1, a recognized inhibitor of v-ATPase located on the lysosomal membrane, which reduces the concentration of H^+^ in lysosome. We exposed the senescent cells to 20 nM Baf-A1 and 20 μM SGJ for 24 h, then, incubated the cells with Lysosensor™ Green DND-189 and photographed under the confocal microscopy. The Lysosensor™ Green DND-189 reagents exhibit a pH-dependent increase in fluorescence intensity upon acidification. We found that the red fluorescence nearly disappeared in the control cells, while it further decreased in the Baf-A1 treated cells and obviously increased in the SGJ treated cells (Fig. 2b). The results indicated that SGJ could increase the concentration of H^+^ in lysosome in the senescent BMSCs. Immunoblotting of LAMP1 (lysosome-associated membrane protein 1) and LAMP2 (lysosome-associated membrane protein 2), the established biomarkers of the function of lysosome, showed a decreased level of these markers in senescent cells. Treating senescent BMSCs with SGJ significantly increased the protein expression of LAMP1 and LAMP2, which restored the lysosomal activity (Fig. 2c). Our results indicated that SGJ might have a protective function on damaged lysosomes.

### SGJ inhibited cultured BMSC senescence and promoted BMSC proliferation

**Fig. 3:**
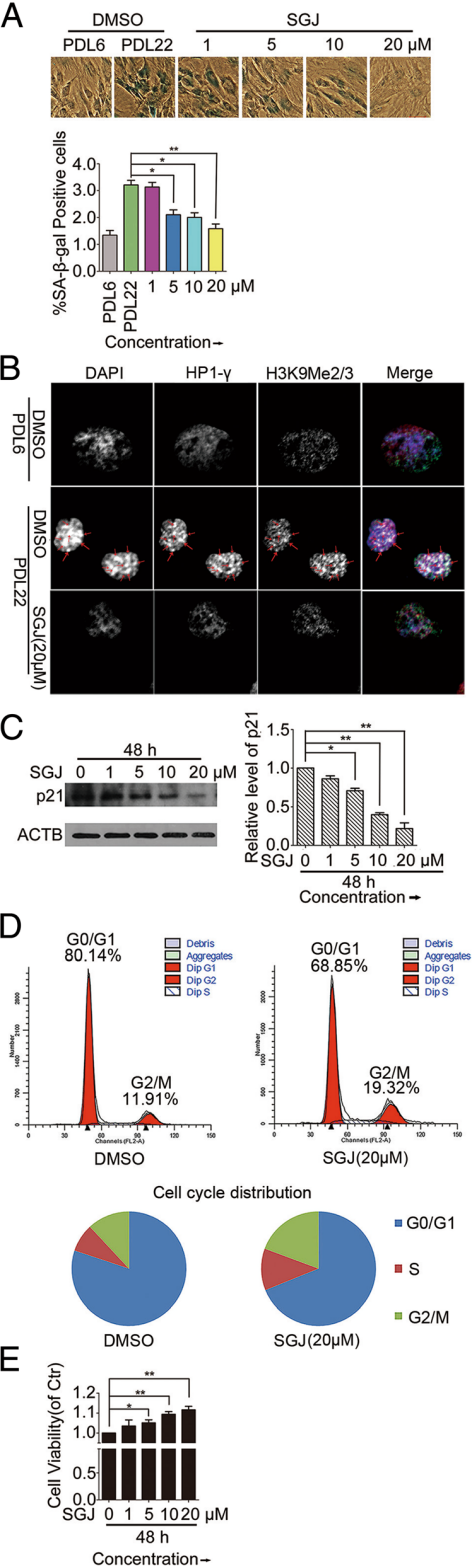
Changes of senescence-associated markers showed that SGJ suppressed cellular aging and promoted cell proliferation. **a** SA-β-gal activity and morphology of young (PDL 6) and senescent (PDL 22) BMSCs. Normal BMSCs at PDL 6 and PDL 22. Senescent BMSCs treated with 1, 5, 10, and 20 μM SGJ for 48 h. Percentage of SA-β-gal-positive cells. **b** Immunofluorescence assay of SAHF and its components HP1-γ and H3K9Me2/3 in young (PDL 6) and senescent (PDL 22) BMSCs treated with/without SGJ (20 μM) for 48 h. The arrow indicating dots are those co-localize with corresponding dots in different channels. **c** Western blot assay of p21 indicated a decline of senescent protein by treating with 1, 5, 10 and 20 μM SGJ for 48 h in senescent BMSCs. **d** Effect of treatment with SGJ (20 μM) for 48 h on proliferation of senescent BMSCs (PDL 22) analyzed by flow cytometry. **e** Cell viability in BMSCs at PDL 22 with 1, 5, 10 and 20 μM SGJ for 48 h. (*, *p* < 0.05; **, *p* < 0.01, results were expressed as means ± SEM, *n* ≥ 3)

We then discovered that SGJ treatment significantly suppressed the senescent biomarkers in BMSCs (Fig. 3). The SA-β-gal staining result showed that there were more positively stained cells in the senescent group (PDL 22) than in the young group (PDL 6) (Fig. 3a). Meanwhile, we found an obvious morphological change in senescent BMSCs. Comparing with the young cells’ spindle-type morphology, the morphology of the senescent BMSCs showed a more flat and spread out status, and the phenomenon of podia and actin stress filaments was increased [32]. We found that using SGJ to treat the senescent BMSCs can significantly decrease the number of SA-β-gal positive cells compared to the control group (PDL 22) (Fig. 3a). SGJ-treated senescent BMSCs exhibited a thinner and smaller morphology. The cytoplasmic extensions and the actin filaments were decreased. The cytoplasmic extensions and the actin filaments were decreased. Senescence-associated heterochromatin foci (SAHF) are specialized domains of facultative heterochromatin related to the contribution to silencing of proliferation-promoting genes in senescent cells. SAHF and its components such as heterochromatin protein 1 (HP1) proteins and lysine 9 di-or tri-methylated histone H3 (H3K9Me2/3) are always used as markers for senescent cells [33]. We found that, compared to the young cells (PDL 6) there were robust punctate DAPI foci in the senescent BMSCs (PDL 22), which co-localize with both HP1 gamma homolog (HP1-γ) and lysine 9 di-or tri-methylated histone H3 (H3K9Me2/3). However, treatment with SGJ for 48 h decreased the punctate DAPI foci and its co-localization with both HP1-γ and H3K9Me2/3 in senescent BMSCs (PDL 22) (Fig. 3b). We also found that SGJ treatment significantly decreased the protein level of p21, an established marker of aging in senescent BMSCs (Fig. 3c).

Next, to find out effects of SGJ on the senescent BMSCs’ growth kinetics, cells (PDL22) were treated with SGJ for 48 h and then stained with propidium iodide (PI) and analyzed by flow cytometry (Fig. 3d). We found about 80.14% senescent BMSCs (PDL 22) stayed at G0/G1 phase and about 19.86% stayed at S phase or G2/M phase (Fig. 3d), while in SGJ-treatment group, the percentage of cells staying at G0/G1 phase decreased to about 68.85% and cells at S phase or G2/M phase increased to 31.15% (Fig. 3d). Also, we found that SGJ could significantly increase the viability of senescent BMSCs (Fig. 3e). Besides, the density of BMSCs increased significantly with the morphology of BMSCs becoming thinner and plumper than control group (PDL 22) (Additional file 1: Figure S1). Taking all these results into account, we can draw a conclusion that SGJ suppresses BMSCs aging in vitro, and promotes the senescent BMSC proliferation. Meanwhile, autophagy plays an important role in the mechanisms of cellular aging. Recently, it is exemplified by studies revealing that increased autophagy flux is a potential mechanism for extending life-span in yeast, drosophila and mouse models. Thus, we investigated the effects of SGJ on BMSC autophagy. We found that SGJ increased the level of protein LC3B significantly and reduced the p62/SQSTM1 protein level by promoting autophagic degradation. To validate the effect of SGJ on autophagy flux, we used Baf-A1/chloroquine (CQ) to block the flow of autophagy (Additional file 1: Figure S2). The data showed that SGJ promoted the flow of autophagy in BMSCs (Additional file 1: Figure S2). It also revealed that treating BMSCs with SGJ could induce cell autophagy significantly.

### SGJ protected and enhanced lysosomal activity via protecting the V0 proton channel of v-ATPase

**Fig. 4:**
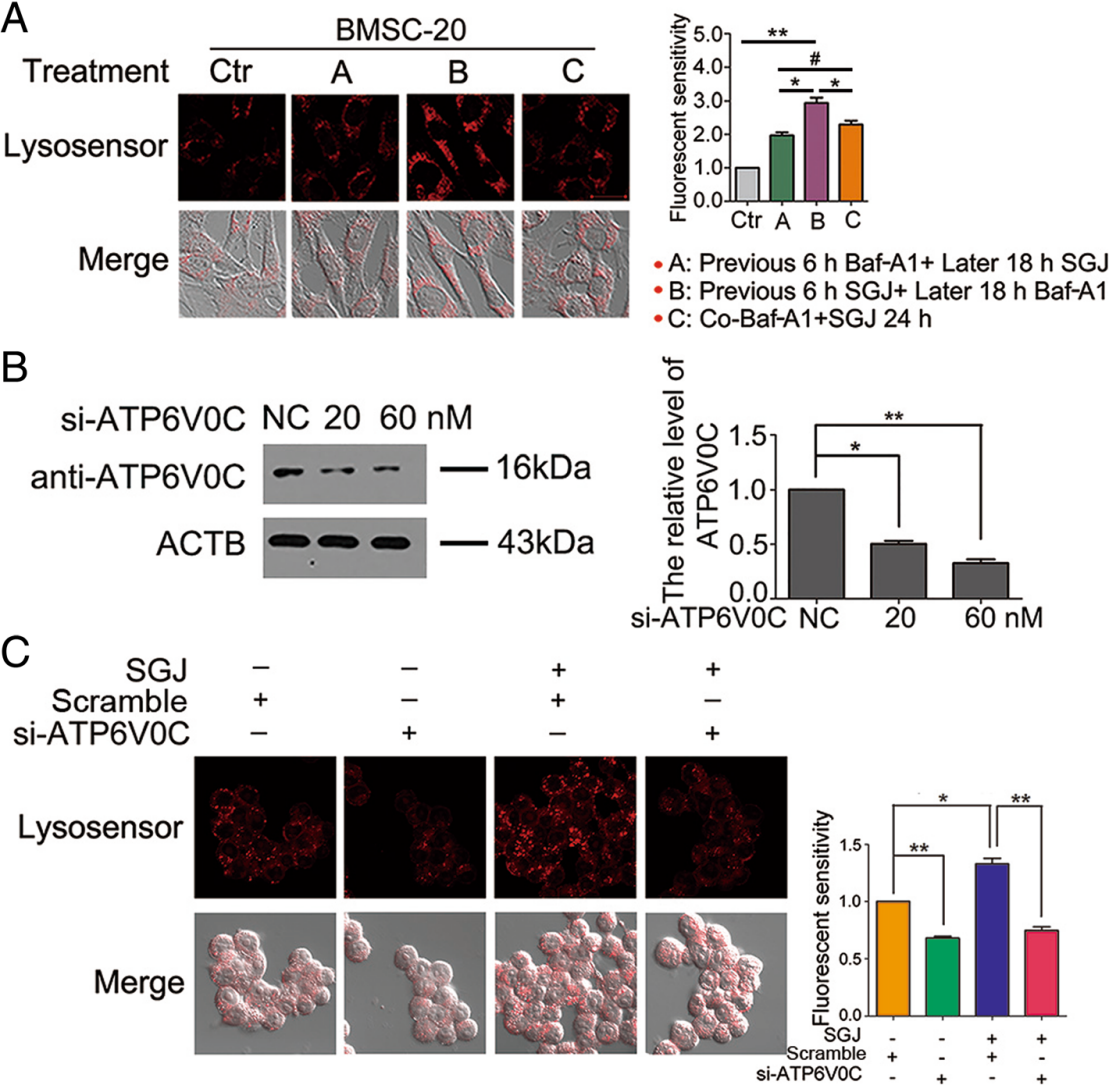
Effects of treatment with SGJ and/ or Baf-A1 and transfection with si-ATP6V0C on lysosomes. **a** Adding 20 nM Baf-A1 and /or 20 μM SGJ in a different order for the designated time. Detect the red fluorescence. **b** Western blot analysis of anti-ATP6V0C protein levels after transfection with si-ATP6V0C for 24 h. **c** 293 T cells were transfected with si-ATP6V0C at 60 nM or scramble RNA for 24 h followed by incubation with 20 μM SGJ for 24 h. Detecting the red fluorescence, and quantification. (#, > 0.05; *, *p* < 0.05; **, *p* < 0.01, results were expressed as means ± SEM, *n* = 3)

The vacuolar ATPase (v-ATPase) is a macromolecular complex. It is responsible for pumping protons (H^+^) into lysosomes and lowering intraluminal pH to the acidic range, and it is needed to activate dozens of hydrolases with acidic pH optima in lysosomes [23]. We have already discovered that SGJ raised the concentration of H^+^ in lysosome in the senescent BMSCs. We wondered if the SGJ-increased concentration of H^+^ in lysosome was a consequence of protecting v-ATPase. The Baf-A1 inhibition site localizes to the V0 proton channel and requires residues of subunit C [34–36]. We next utilized Baf-A1 and / or SGJ to explore the targeting sites of SGJ. We designed three typical experiments: (A) treatment with 20 nM Baf-A1 for 6 h and then treatment with 20 μM SGJ for 18 h; (B) treatment with 20 μM SGJ for 6 h and then treatment with 20 nM Baf-A1 for 18 h; (C) co-treatment with 20 nM Baf-A1 and 20 μM SGJ to the senescent BMSCs for 24 h. Next, we detected the red fluorescence intensity by Lysosensor™ Green DND-189 staining (Fig. 4a), imaged the cells’ morphologic changes and calculated the cell viability by SRB (sulforhodamine B assay) (Additional file 1: Figure S3). We found that the experimental group pretreating with 20 μM SGJ showed a stronger red fluorescent intensity, thinner cells’ morphology and higher cell viability in the presence of Baf-A1. The results indicated that SGJ might occupy the action sites of Baf-A1 competitively, which targeted the V0 proton channel of v-ATPase to protect its activity, resulting in increasing the concentration of H^+^ and preventing the destructive effect of Baf-A1 in lysosome. For further exploring the possible molecular mechanism of SGJ in increasing the concentration of H^+^, 293 T cells were transfected with si-ATP6V0C at 20 or 60 nM for 24 h. Si-ATP6V0C could markedly decrease the activity of the V0 proton channel of v-ATPase. The efficiency of ATP6V0C knockdown was verified (Fig. 4b). The fluorescent intensity results showed that si-ATP6V0C decreased the activity of v-ATPase and SGJ increased the concentration of H^+^ (Fig. 4c).

**Fig. 5:**
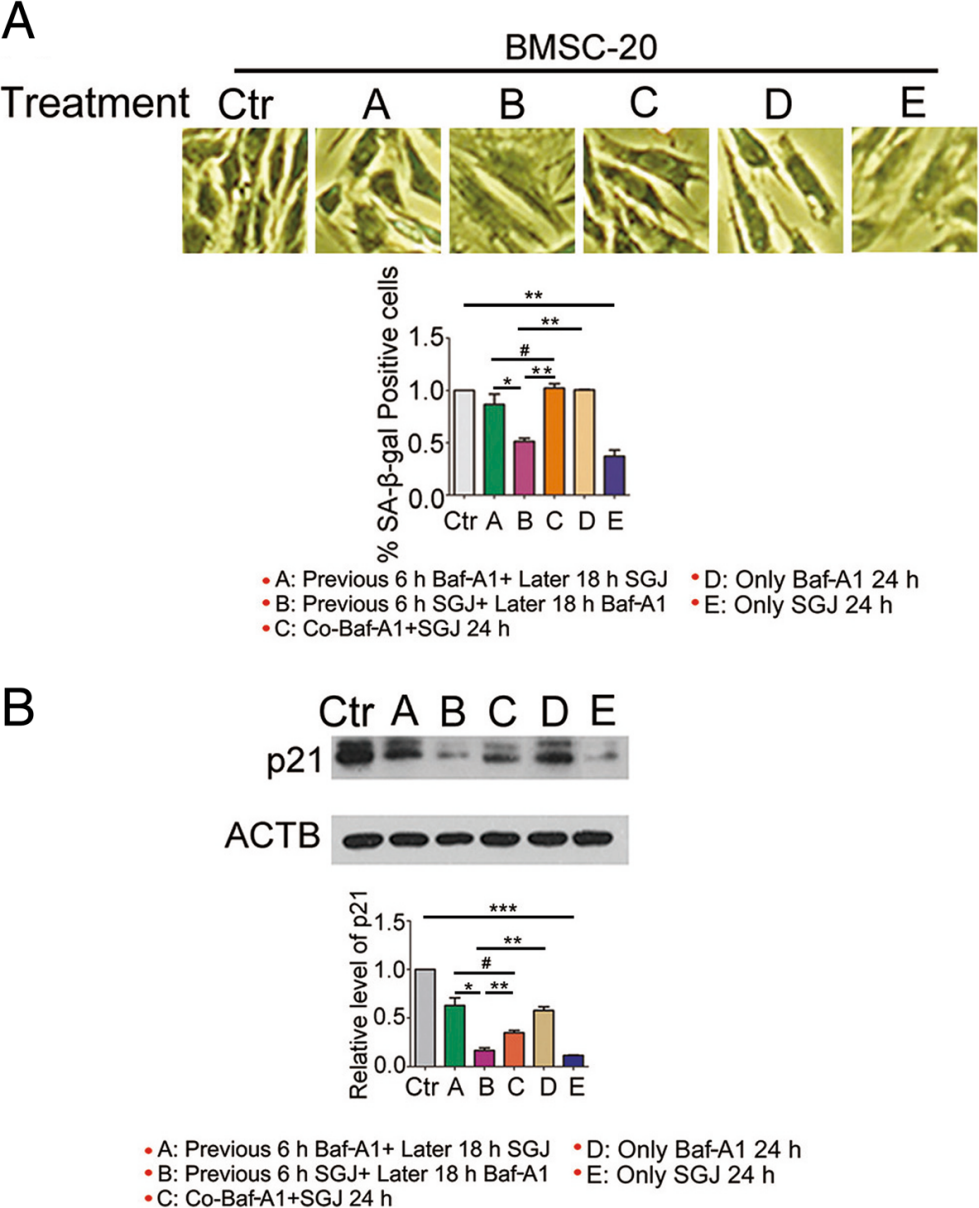
Changes in SA-β-gal positive cells and p21 protein level after treatment with SGJ and/or Baf-A1. **a** BMSCs were treated as the above condition. Senescent cells were stained blue under a phase-contrast microscopy. The percentage of positively stained cells was estimated by counting at least 1500 cells for each sample. **b** Western blot analysis of p21 with β-actin as a loading control. (#, > 0.05; *, *p* < 0.05; **, *p* < 0.01; ***, *p* < 0.001, results were expressed as means ± SEM, *n* = 3)

Next, to further prove the targeting sites of SGJ is the V0 proton channel of v-ATPase. We added SGJ and / or Baf-A1 in a different order and detected the changes in SA-β-gal positive cells as well as the p21 protein level. Compared with other experimental groups, we found that the experimental group pretreating with 20 μM SGJ showed a fewer SA-β-gal positive cells and lower p21 protein level in the presence of Baf-A1 (Fig. 5). The data revealed that SGJ is able to occupy the action site of Baf-A1 preferentially and prevent cell senescence effectively.

## Discussion

In the process of cell aging, the lysosomal activity is decreased and the concentration of H^+^ in lysosome is reduced [1, 28, 37]. Meanwhile, the autophagic ability of the senescent cells is reduced [13, 38, 39]. In this study, we discovered that the novel small molecule, 3-butyl-1-chloro imidazo [1, 5-a] pyridine-7-carboxylic acid (SGJ) could co-locate with lysosome and protect its function. We also found that SGJ could bind to v-ATPase, promote the inflow of H^+^ in lysosomes and inhibit the decrease in the concentration of H^+^ in senescent BMSCs. LAMP1 and LAMP2 are functional proteins on the lysosomal membrane, and they have been linked to protection of membrane from degradation by lysosomal hydrolases [40]. However, both the lysosomal activity and the protein levels of LAMP1 and LAMP2 decreased in the process of cell aging [40, 41]. Protein levels of LAMP1 and LAMP2 increased after treatment with SGJ, showing that SGJ protected the lysosomal integrity and function. The cellular lysosomes maintain a low acidic lumen by means of the proton pump and v-ATPase [21]. With the acidic pH optimum conditions, dozens of hydrolytic enzymes in the lysosome are activated [23]. However, new reports implicate lysosomal pH dysregulation in cellular aging [1]. Therefore, an abnormal rise in lysosomal pH can have significant effects on lysosomal digestion, for example, strongly inhibiting hydrolases with the most acidic pH optima, or potentially elevating other hydrolases’ activities with pH optima being closer to neutral [23]. SGJ can promote the function of lysosomes themselves by elevating activities of hydrolases with the most acidic pH optima. Thus, this indicates that the functions of lysosomes restored and the levels of cell autophagy are elevated in the senescent cells under treatment with SGJ.

In fact, to promote the efficiency of BMSCs for clinical therapies and tissue engineering, we should pay attention to both intrinsic aging of BMSCs in vivo and their aging in vitro when these cells may require expansion in vitro before use as well as for future research [42]. It is no doubt that there exist differences between PDL-based aging model in vitro used in our research and those naturally senescent BMSCs in vivo. For instance, in vivo, stem cell fate and activities are largely determined by their microenvironment. However, when cultured in vitro without the presence of regulatory cues which exist in vivo, MSCs may have different functions and properties [43]. When viewed from this perspective, whether SGJ could exert the same effect on those BMSCs aged in vivo should be investigated further. However, many studies indicated that elements changed during aging, including autophagy, lysosomal function and activity, and v-ATPase activity, were not only found in vivo [1, 15, 38, 44] but also in vitro [14, 15, 44–46]; therefore, according to our work, it is reasonable to draw a conclusion that SGJ is a potential lead compound for suppressing BMSC senescence. At least, it could be used as a powerful tool for studying and understanding the mechanism of aging.

## Conclusions

**Fig. 6:**
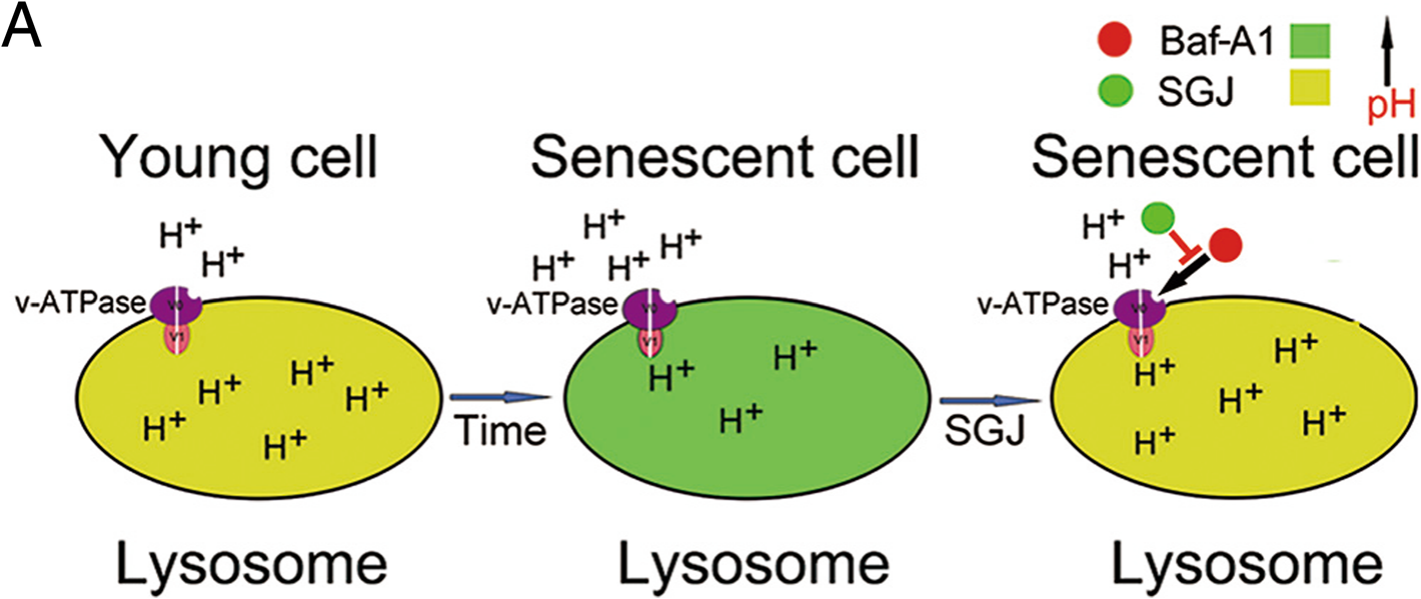
Schematic presentation of SGJ preventing the disorders of lysosomal acidification via v-ATPase in senescent BMSCs. **a** In the process of cell aging, the lysosomal activity is decreasing and the concentration of H^+^ in lysosome is reducing. SGJ can bind to v-ATPase, promote the inflow of H^+^, and inhibit the decrease of the concentration of H^+^ in senescent lysosomes

Our work showed that through treating the senescent BMSCs with SGJ, the condition of cell aging is inhibited and the viability of the senescent cells is improved. SGJ is a potential lead compound for suppressing BMSCs senescence. In addition, we further explored the targeted site of SGJ. By adding SGJ and/or Baf-A1 in a different order, we found that SGJ could target and occupy the V0 proton channel of v-ATPase, indicating that SGJ and Baf-A1 have a competitive relationship and SGJ protects the v-ATPase activity via preventing the destructive effect of Baf-A1 on lysosome (Fig. 4c). The results suggested that SGJ was a new Baf-A1 antagonist that promoted autophagy and inhibited senescence in bone marrow-derived mesenchymal stem cells (Fig. 6).

## Additional file

Additional file 1: Supplementary figures are included in it. (DOC 1364 kb)

## Abbreviations

AO: Acridine orange
Baf-A1: Bafilomycin-A1
BMSCs: Bone marrow-derived mesenchymal stem cells
CQ: Chloroquine
DMEM-LG: Dulbecco’s modified Eagle’s medium-low glucose
DMSO: Dimethylsulfoxide
H3K9Me2/3: Lysine 9 di-or tri-methylated histone H3
HP1: Heterochromatin protein 1
HP1-γ: HP1 gamma homolog
LAMP1: Lysosome-associated membrane protein 1
LAMP2: Lysosome-associated membrane protein 2
LC3B: MAP1LC3
p62: SQSTM1
PBS: Phosphate-buffered saline
PDL: Population doubling level
PI: Propidium iodide
PVDF: Polyvinylidene fluoride
SAHF: Senescence-associated heterochromatin foci
SA-β-gal: Senescence-associated beta-galactosidase staining
SGJ: 3-Butyl-1-chloro imidazo [1,5-a]pyridine-7-carboxylic acid
si-ATP6V0C: Small interfering RNA
SRB: Sulforhodamine B assay
TCA: Trichloroacetic acid
v-ATPase: Vacuolar H^+^-ATPase
