# Supplementary material for: A pH probe inhibits senescence in mesenchymal stem cells
Source: Stem Cell Res Ther. 2018 Dec 7;9:343. doi: 10.1186/s13287-018-1081-0 (PMC6286523; doi:10.1186/s13287-018-1081-0)
Supplement: Supplementary file 1 — Supplementary figures are included in it. (DOC 1364 kb) [file 13287_2018_1081_MOESM1_ESM.doc]

**Additional file 1 for**

**A pH Probe Inhibits Senescence in Mesenchymal Stem Cells**

Lihong Wang ^[1] #^, Xianjing Han^[1] #^, Guojing Qu^[1]^, Le Su^[1]^, Baoxiang Zhao*^[2]^ and Junying Miao*^[1], [3]^

**Supplemental Figures and Figure legends:**


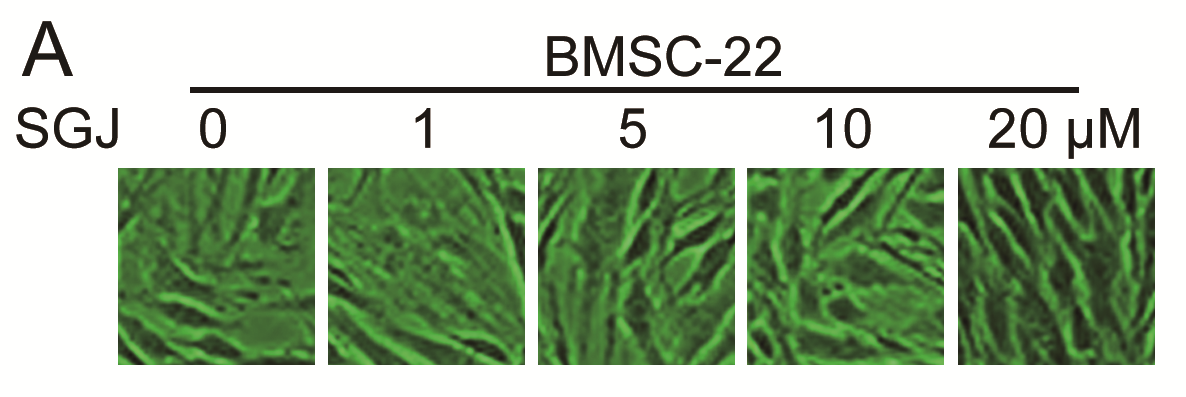


**Figure S1.** (A) SGJ-treated senescent BMSCs exhibited a thinner and smaller morphology.


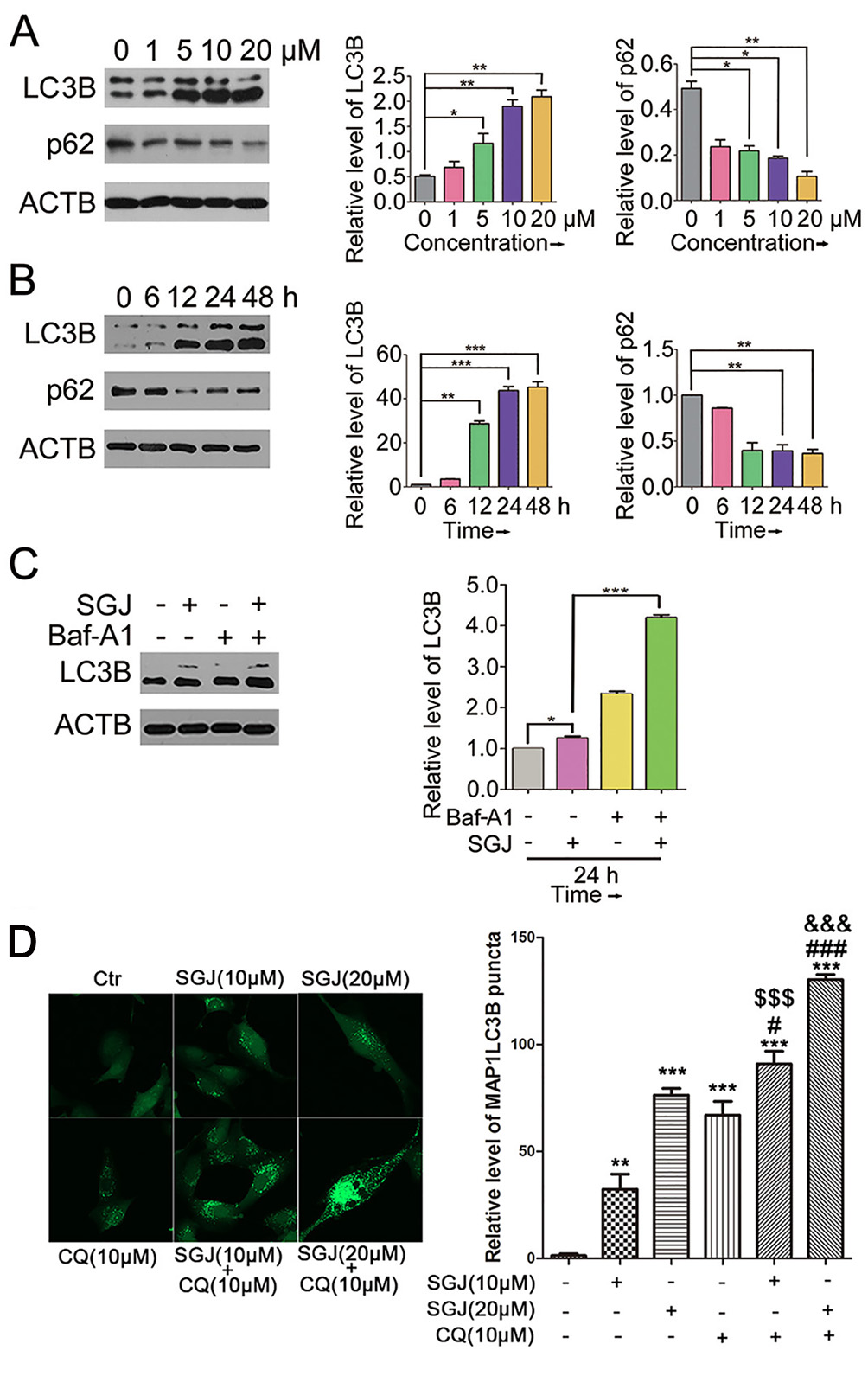


**Figure S2.** SGJ-induced cell autophagy. (a) Western blot analysis of LC3B and p62 (*, p < 0.05; **, p < 0.01, results were expressed as means ± SEM, n=3). (b) Western blot analysis of LC3B and p62 (**, p < 0.01; ***, p < 0.001, results were expressed as means ± SEM, n=3). (c) Western blot analysis of LC3B (*, p < 0.05; ***, p < 0.001, results were expressed as means ± SEM, n=3). (d) Immunofluorescence of GFP-LC3B puncta in U87 cells. (“ * ” VS. Control; **, p < 0.01; ***, p < 0.001; “ # ” VS. CQ (10μM); #, p < 0.05; ###, p < 0.001; “ $ ” VS. SGJ (10μM); $$$, p < 0.001; “ & ” VS. SGJ (20μM); &&&, p < 0.001, results were expressed as means ± SEM, n=3).

**
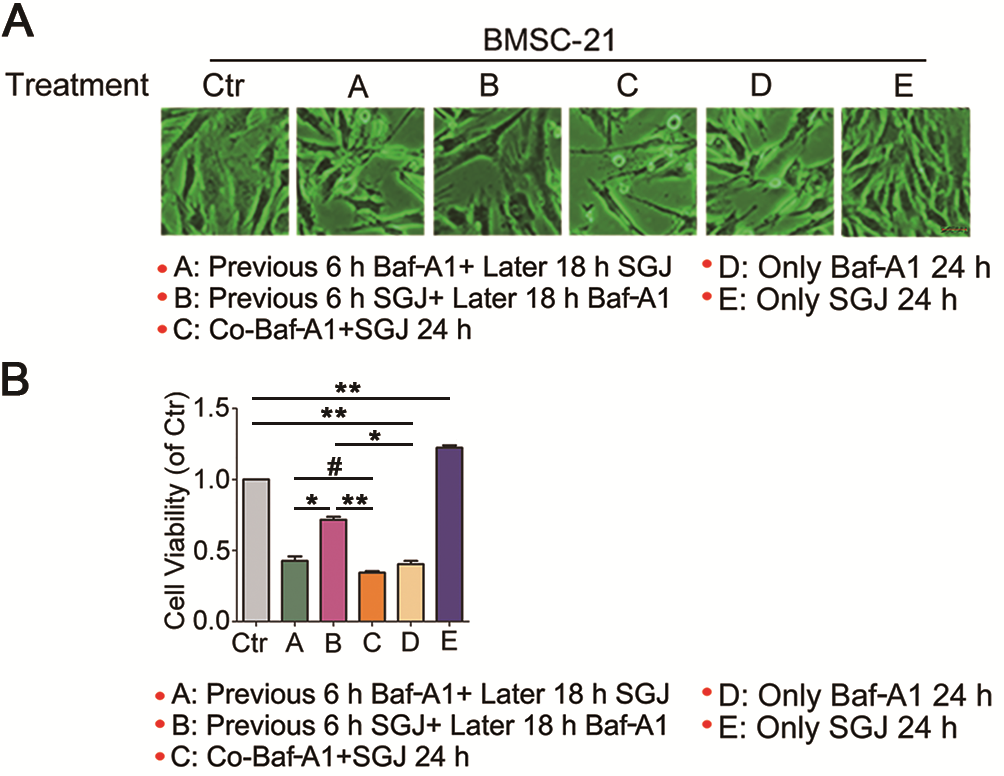
**

**Figure S3.** Effects of treatment with SGJ and / or Baf-A1 in a different order on cell morphology (A) and cell viability (B) in BMSCs (#, p> 0.05; *, p < 0.05; **, p < 0.01, results were expressed as means ± SEM, n=3).
